# Psychometric evaluation of the postpartum specific anxiety scale in an Iranian population (PSAS-IR)

**DOI:** 10.1186/s12884-021-04085-w

**Published:** 2021-09-04

**Authors:** Robab Hasanzadeh, Mohammad Asghari Jafarabadi, Shirin Hasanpour, Victoria Fallon, Sergio A. Silverio, Reyhane Montazeri, Mojgan Mirghafourvand

**Affiliations:** 1 Department of Midwifery, Bonab Branch, Islamic Azad University, Bonab, Iran; 2 Department of Statistics and Epidemiology, School of Medicine, Zanjan University of Medical Sciences, Zanjan, Iran; 3 Center for the Development of Interdisciplinary Research in Islamic Sciences and Health Sciences, Tabriz University of Medical Sciences, Tabriz, Iran; 4 Women’s Reproductive Health Research Center, Tabriz University of Medical Sciences, Tabriz, Iran; 5 Department of Psychology, Institute of Population Health, University of Liverpool, Liverpool, UK; 6 Department of Women & Children’s Health, School of Life Course Sciences, King’s College London, London, UK; 7 Student Research Committee, Tabriz University of Medical Sciences, Tabriz, Iran; 8 Social determinants of Health Research Center, Tabriz University of Medical Sciences, Tabriz, Iran

**Keywords:** Postpartum anxiety, Psychometric, Iran

## Abstract

**Background:**

Anxiety is one of the most prevalent mental health disorders among mothers during the postpartum period, which can lead to maternal and infant physical and psychological consequences. The Postpartum Specific Anxiety Scale (PSAS) predicts unique variance in postnatal outcomes over and above general anxiety tools. It has never been used in Iran and its validity and reliability have not been assessed either. Therefore, the present study aimed to translate and investigate the psychometric properties of the PSAS-IR.

**Methods:**

510 women, from six weeks to six months postpartum, were selected through random sampling in 2020. After forward and back-translation, the face validity, content validity, and construct validity of PSAS (through confirmatory factor analysis) were examined. The reliability of the scale was assessed using both internal consistency (Cronbach’s alpha) and test-retest stability methods.

**Results:**

CVI and CVR values of the PSAS tool were 0.89 and 0.88, respectively. The good fit indices confirmed the validity of four-factor structure. Cronbach’s alpha coefficient and Intra Correlation Coefficient (ICC) equaled 0.93 and 0.92, respectively.

**Conclusion:**

The Persian version of PSAS is a valid and reliable four-factor scale, it will improve the measurement of postpartum anxiety in an Iranian setting. This will improve the measurement of postpartum anxiety in an Iranian setting.

## Background

The birth of an infant causes numerous long-term changes; some of these changes are accompanied by stress and anxiety. These anxieties include concerns about body image, gaining weight, the health of the infant, interpersonal relationships, and infant care [1–4].

Postpartum anxiety is one of the prevalent mental problems among mothers, which occurs more than postpartum depression, and if it is not treated, it can increase the risk of postpartum depression. The prevalence of anxiety disorder in the first year after birth is between 9.9 and 20% [5, 6]. Anxiety can affect a mother’s maternal abilities by restricting her activities and causing irrational fears [7]. Also, studies indicate that a mother’s anxiety can prevent optimal mother-child interactions, impact upon infant feeding outcomes and behaviours, and hinder the socio-cognitive development of the child [7–10].

In these studies, postpartum anxiety in women was measured using general tools such as Spielberger State-Trait Anxiety Inventory [11]. These were designed and validated for use in general populations and therefore contain items that are inappropriate to the period after birth (e.g. I feel rested). A body of evidence now demonstrates use of tools pertinent to the period of childbearing are more acceptable to postpartum women and have better predictive utility than general anxiety measures in predicting perinatal outcomes [2, 12–14] due to their assumed ability in determining small but considerable clinical changes [15].

PSAS was designed by Fallon et al., comprising 51 questions as a 4-point Likert scale to measure the frequency of maternal and infant focused anxieties. There are four subscales: maternal competence and attachment anxieties; infant safety and welfare anxieties; practical infant care anxieties; and psychosocial adjustment to motherhood. Maternal competence and attachment anxieties subscale have 15 items for example “I have had negative thoughts about my relationship with my baby” and “I have felt that my baby would be better cared for my someone else”. Infant safety and welfare anxieties subscale have 11 items for example “I have worried about my baby being accidentally harmed by someone or something else” and “I have repeatedly checked on my sleeping baby”. Practical infant care anxieties subscale have 7 items for example “I have worried about my baby’s milk intake” and “I have worried about my baby’s weight”, and also psychosocial adjustment to motherhood subscale have 18 items for example “I have felt resentment towards my partner” and “I have felt tired even after a good amount of rest” [2].

The psychometric work conducted in English speaking samples demonstrates the validity, reliability, and predictive utility of the measure in high income settings. In Duran’s study, the psychometric properties of Turkish version of the PSAS has been evaluated and it was found that it could be used as a valid and reliable tool in Turkish women [16]. However, neither validity nor reliability of this scale has been assessed in a middle income setting, such as Iran. A scale that has been developed in an English-speaking society can be used in the other population with cultures other than the one(s) for which they were originally developed if the validity of translation into other languages is determined. Also, even if items can be literally translated, it is important to consider whether the items are meaningful and are being interpreted similarly across cultures [17]. Therefore, the present research aimed to translate and investigate psychometrics properties of an Iranian version of the PSAS [PSAS-IR].

## Methods

### Study participants

The participants of this research were women who recently gave birth at term from six weeks to six months postpartum. The other inclusion criteria were: singleton pregnancy, having term birth with a healthy infant more than 2.5 Kg, giving birth vaginally or cesarean section and self-reported physical health. The exclusion criteria were: history of mental illness according to maternal self-report, history of traumatic event in the family in the past six months. Mental illness and post-traumatic stress disorder (PTSD) can affect postpartum specific anxiety symptoms [18, 19], therefore women with anxiety disorders or PTSD were excluded.

### Sample size

Literature suggests that the sample size required for factor analysis is 5 to 10 participants per item [20]. Considering 51 items and 5 participants per item, a sample of 255 women was required. However, due to cluster sampling and by applying the design effect equal to 2, the sample size was increased up to 510 (*n* = 510).

### Translation process

After gaining the necessary permissions from the PSAS working group, the original version was translated from English into Persian using approved translation methods [21]. This was done by a native translator with good command of the Persian language. The translated version was reviewed by the research team. Then, the version mentioned in the previous step was back-translated from Persian into English by two proficient back-translators who did not participate in the previous step. Then, two people familiar with the specialized concepts and with good command of both languages reviewed the translated and back-translated versions and a final version was decided upon.

### Data collection

Multistage random sampling was carried out. First, using a computer program (www.Random.org), half of the health centers (80 centers) were randomly selected in the city of Tabriz (cluster sampling). Then, among the 50% of centers selected randomly, a list of mothers with six weeks to six months postpartum were extracted, the number of the participants selected from each center was then determined proportionally, and the mothers were selected randomly from the list. Then, a telephone call was made to the selected mothers and the explanations were provided regarding the reasons and methods of conducting the research. Maternal statuses were examined to see whether they met the eligibility criteria. Participating mothers were requested to be at the health center at a determined time. During the in-person meeting, written informed consent was obtained from the mothers and a socio-demographic and obstetrics characteristic questionnaire along with the PSAS-IR were completed through interviews with the participants. The PSAS is a self-reported measure but we selected interview for completing questionnaire to enable illiterate women to participate in this study.

In some days of data collection period, there was extensive lockdowns in Iran because of coronavirus. Therefore, sometimes we had to stop data collection. We requested participants for being in health centers with face mask as well as the questionnaire was completed for each participant in a room in health centers where nobody was there and we tried to choose a day for the mother attendance in the health center that coincides with the time of infant’s care or vaccination.

### Data analysis

Statistical analysis was performed in SPSS version 24 and Amos version 24. Normality of the distribution of the PSAS items checked before conducting the analyses and all items have normal distribution.

### Face and content validity

To determine the face validity of the scale, 30 women were randomly selected and asked to assess the questions with respect to their difficulty, appropriateness, and ambiguity. Then, the impact score for each item was calculated using the equation: Impact Score = Importance (average of the answers given to an item) x Frequency (number of answers to option 4), on the basis of which the responses were calculated using the Likert scale from score 1 (totally difficult or not clear) to 4 (totally easy or clear). In the case of obtaining a score less than 1.5, that item was deleted [22].

The content validity was conducted both qualitatively and quantitatively. In the qualitative component, ten specialists across midwifery, reproductive health, and psychiatric nursing were asked to examine and provide corrective opinions on the translation of each question with respect to its grammar, appropriateness of wording, and the sentence structure. In the quantitative component, content validity ratio (CVR) and content validity index (CVI) were both calculated. To determine the content validity index of the questions were assessed regarding relevance, clarity, and simplicity based on a 4-point Likert scale.

### Confirmatory factor analysis

Confirmatory factor analysis (CFA) was used to assess the model fit of the factors. A good fit of the indices was employed for the assessment of the proportion of the model. For the approval of the model, Root Mean Square Approximation (RMSEA) less than 0.08, Error Approximation Square Mean Square Root Standardized (SRMSEA) < 0.08, Index Fit Index (CFI), Adjusted Goodness-of-Fit Index (AGFI), Incremental Fit Index (IFI) and Relative Fit Index (RFI) ≥ 0.90, Index Tucker-Lewis (TLI), Normed Fit Index (NFI ≥ 0.95), Normed (× 2 / df) < 5.0 crystal were all considered. Also, the significance of the model coefficient test and the correlation test between the factors were examined in the CFA.

### Reliability

To determine the reliability of the PSAS-IR, a Cronbach’s alpha test was carried out on the full sample and the alpha coefficient (internal consistency) was determined. Test-retest reliability was carried out on a sub sample of 20 participants after two weeks and intra-correlation coefficient (ICC) was derived.

## Results

### Participants characteristic

The full possible sample was 1450 women in 40 health centers; 510 women selected randomly. A majority of eligible women (96%) agreed to participate in the study. From April 2020 to December 2020, mothers entered the research. The mean (standard deviation) participant age was 29.47 (4.9) and more than three-fourth (79.8%) of them were housewives. Other socio-demographic characteristics of the participants are provided in Table 1.

**Table 1:** Characteristics of the study participants (*n* = 510)

Characteristics	N (%)
Age (Years)^a^	29.47 (4.9)
Education	
Intermediate or below	24 (4.7)
Diploma and high School	124 (24.3)
University	362 (71)
Job	
Housewife	407 (79.8)
Employee	103(20.2)
Income	
Not at all sufficient	24 (4.7)
Relatively sufficient	279 (54.7)
Completely sufficient	207 (40.6)
Mode of delivery	
Vaginal	183 (35.9)
Caesarean section	327 (64.1)

^a^The numbers were reported as mean (standard deviation)

### Face and content validity

In the face validity examination, all items were described as suitable, without any ambiguity and difficulty and the lowest score was 1.5. Furthermore, in examining the content validity, all items achieved the minimum acceptable amount of CVI and CVR. The CVR of the scale equaled 0.88 and the CVI equaled 0.89. Item-CVI (I-CVI) for all 51 items was 0.8 to 1.

### Reliability

The Cronbach’s alpha coefficient was calculated to be 0.93 for this scale, which is an indication of the desired internal correlation of the scale. Item 15 (I have felt that I should not need help to look after my baby) and Item 46 (I have worried about returning to work) were deleted due to a Cronbach’s alpha value less than 0.3. In the test-retest method, the ICC for the scale was obtained 0.92. Cronbach’s alpha coefficients were calculated 0.87 for “practical infant care anxieties” subscale, 0.93 for “maternal competence and attachment anxieties” and “infant safety and welfare anxieties” subscales and 0.94 for “psychosocial adjustment to motherhood” subscale. Also, ICC was calculated 0.89 for two subscales “practical infant care anxieties” and “psychosocial adjustment to motherhood”, 0.92 for “maternal competence and attachment anxieties” and 0.95 for “infant safety and welfare anxieties” subscale.

### Construct validity

**Fig. 1:**
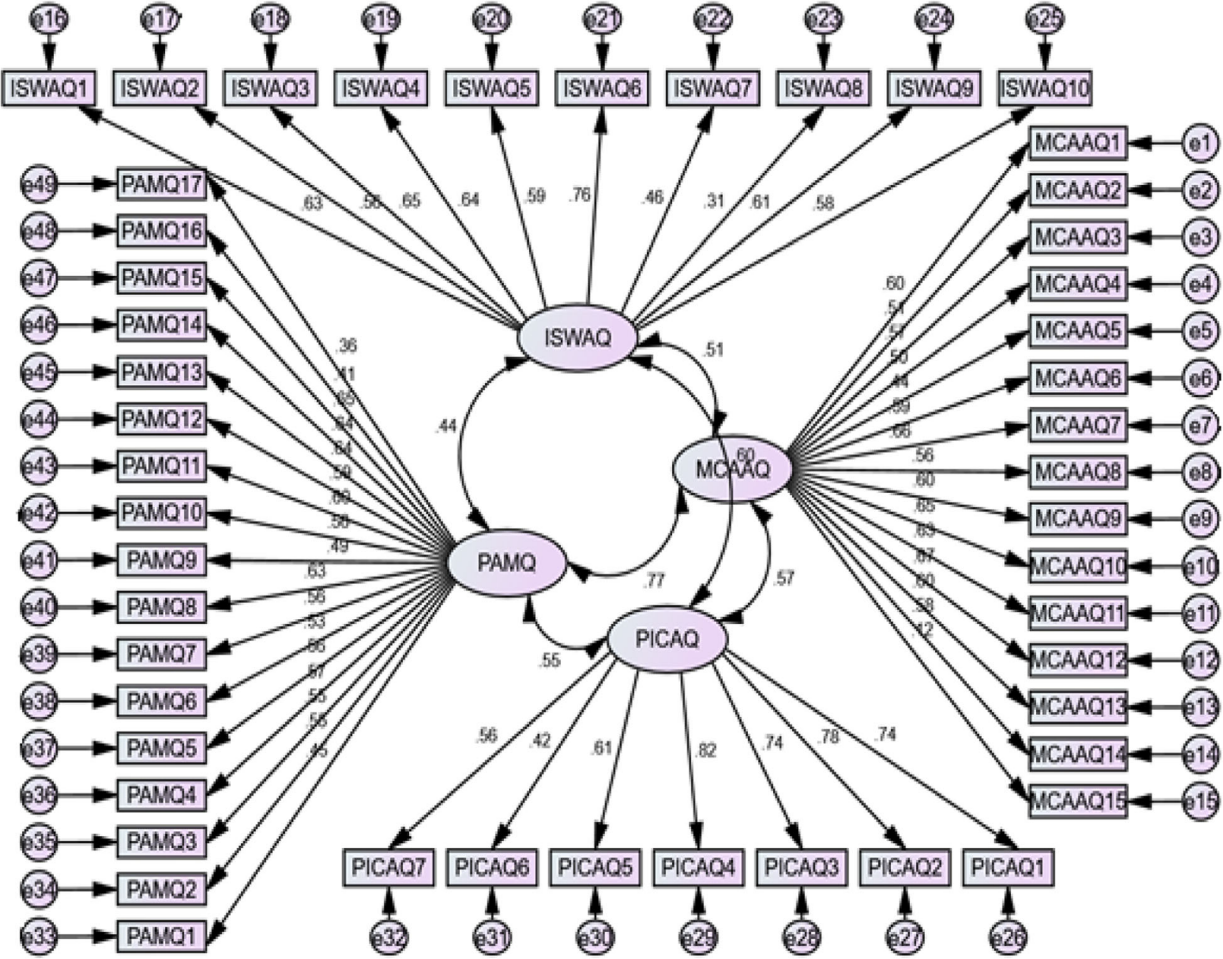
CFA Factor Loading

In the CFA, the obtained x^2^/df and RMSEA were 2.237 and 0.049 which confirmed the validity of the four-factor model. Furthermore, according to good fit indices, FI (0.99), AGFI (0.91), NFI (0.96), RFI (0.94), IFI (0.99), and CFI (0.99) this model achieved a desirable level of fit, and accordingly, good construct validity. A Path diagram with standard coefficients of factor analysis is provided as a conceptual model in Fig. 1.

## Discussion

The present research was conducted to determine the psychometric properties of the PSAS for Iranian mothers. It was demonstrated that the Persian version of this scale is a valid tool for the assessment of postpartum anxiety in Iranian mothers. Validity was assessed and confirmed using face validity (qualitative and quantitative), content validity (qualitative and quantitative), and CFA. The reliability of the tool was also examined and approved through internal consistency (Cronbach’s alpha coefficient) and test-retest stability. The factor structure of the PSAS in the present study was almost like to the UK version which demonstrates that the types of anxieties women experience hold constant across income settings.

In comparison with the UK version, in the Iranian version of the PSAS, question 15 concerning the mother’s feelings about the need for no other person to help care for the baby was deleted from the original version, which could be related to cultural differences and their beliefs in child rearing practices. Inadequate supports by the organizations from the employee women have caused the mothers to be worried about their child care after the maternal leave. They had to sometimes assign replacements for themselves such as grandmothers, aunts and etc. [23].

Also, question 46, which was about the mother’s worries about returning to work, was deleted in the Iranian version because it had a Cronbach’s alpha value less than 0.3. This seems understandable given the study sample composition of predominately housewives. 16–20% of women in Iranian context are employee, therefore the sample of this study are representative of the population [24].

The Cronbach’s alpha coefficient for the scale was calculated to be 0.93, which indicates excellent reliability. This is comparable to the UK and Turkish versions of the PSAS where the Cronbach’s alpha coefficient for the total scale was 0.93 and 0.91 [2, 16] and demonstrates the reliability of the tool across diverse settings. The PSAS-IR also demonstrated excellent stability over time with a marginally improved coefficient when compared to the UK version [2].

The PSAS with a four-factor structure including anxiety pertinent to maternal competency and attachment, infant safety and welfare, practical infant care, and psychosocial adjustment to motherhood had a proper face and content validity. Face validity means that items comprehensively covers the different components of anxiety to be measured and content validity indicate that items are sensible, appropriate, and relevant to the people who use the measure [25]. In the Turkish version of PSAS, the scale had a single factor structure and items had the factor loadings in the appropriate range (0.30–0.58) [16].

The factors of this scale matched the results of some other studies. In the research by Phillips et al., 65% of the mothers reported child’s safety and welfare-related anxiety, 53% of mothers reported performing maternal role-related anxiety, and 18% of the mothers reported infant daily care-related anxiety [26]. Highet et al. in a qualitative study examined the women’s experiences regarding pregnancy anxiety and the postpartum period. One of the obtained themes was “adjustment problem”, which included anxiety pertinent to changes of body appearance, daily activities, and social roles [27]. In Wardrop and Papaduik study [28], the participants’ sense of competence, or perceived lack thereof during the postpartum, appeared to have contributed to their sense of anxiety. Also, in the study of Martini et al. [29], worries about the health of child was one of the reasons for development of postpartum anxiety.

The results of the UK study indicated the distinction between postpartum anxiety and depression [2] and also anxiety experienced during other periods of life, which reveals the importance of the specific tool for predicting the postnatal consequences caused by postpartum anxiety [30, 31]. Using the PSAS and distinguishing it from general anxiety and depression can help to screen, predict, and even prevent this anxiety more precisely [31].

Work using the original PSAS demonstrates that it is a more powerful predictor of maternal and infant outcomes than other general tools. Findings of some studies provided evidence to support the predictive utility of the PSAS and demonstrated that higher levels of postpartum specific anxiety were associated with some outcomes such as impaired overall bonding scores and lower odds of breastfeeding exclusively [13, 14], therefore, it is suggested that the predictive validity of the PSAS-IR in longitudinal designs is examined in next steps as well as looking at measurement invariance of the PSAS across different countries where it is currently being used.

### Strengths and limitations

This is the first investigation of the psychometric properties of the PSAS in an Iranian setting. Random sampling and inclusion of women with a vaginal delivery or cesarean section are among other strengths of this research. The main limitation of this study is that the participants in this study consisted of women with singleton and term pregnancy, therefore, the results couldn’t be generalized to women with multiple or preterm pregnancy. Also, women with a history of mental illness in this study were excluded which decreases the generalizability of the results to these women. Some types of validity such as criterion and concurrent validity weren’t assessed in this study. Also, Sampling for this study was carried out during the COVID-19 pandemic that may influence on the results of the present study. Postpartum women reported high levels of anxiety during this pandemic and worries about children and childcare and economic worries were also important factors in women’s anxiety [32].

## Conclusion

This is the first approved translation and adaptation of the PSAS in a Middle-Income Country and it performs in the same manner as the UK tool. The results showed that the PSAS-IR is a valid and reliable tool for the assessment of postpartum anxiety.

## Data Availability

Datasets used and analyzed during this study are available from the corresponding author on reasonable request.

## Abbreviations

AGFI: Adjusted Goodness-of-Fit Index
CFI: Index Fit Index
CFA: Confirmatory Factor Analysis
CVI: Content Validity Index
CVR: Content Validity Ratio
ICC: Intraclass Correlation Coefficient
IFI: Incremental Fit Index
NFI: Normed Fit Index
PTSD: Post-Traumatic Stress Disorder
PSAS: Postpartum Specific Anxiety Scale
PSAS-IR: Postpartum Specific Anxiety Scale –Iranian version
RFI: Relative Fit Index
TLI: Index Tucker-Lewis.
